# Evaluating Acceptance and Use of Digital Health Platforms for Consultation and Medication Delivery Among Men Who Have Sex With Men and Transgender People Living With HIV: Cross-Sectional Survey Informed by an Extended UTAUT Approach

**DOI:** 10.2196/93129

**Published:** 2026-10-06

**Authors:** Emmanuel Oluwatosin Oluokun, Festus Fatai Adedoyin, Huseyin Dogan, Nan Jiang

**Affiliations:** 1 School of Computing and Engineering, Faculty of Media, Science & Technology, Bournemouth University, Fern Barrow, Poole, England, BH12 5BB, United Kingdom, 44 01202524111

**Keywords:** UTAUT, digital health, mHealth, HIV/AIDS, transgender, stigma, technology acceptance, mobile health, Unified Theory of Acceptance and Use of Technology

## Abstract

**Background:**

Digital health platforms can expand access to HIV care, but among men who have sex with men (MSM) and transgender people living with HIV and AIDS in Nigeria, adoption is shaped by structural stigma, criminalization, and fear of disclosure as much as by system functionality. Teleconsultation and medication-delivery platforms offer alternative pathways to care, but their acceptance within marginalized populations cannot be assumed and requires empirical investigation.

**Objective:**

This study aimed to examine the factors influencing behavioral intention (BI) to adopt TechAids, a confidentiality-oriented digital health platform designed to support HIV consultation and service access among MSM and transgender individuals in Nigeria, using an extended Unified Theory of Acceptance and Use of Technology (UTAUT) framework.

**Methods:**

A cross-sectional survey was conducted in Lagos, Nigeria, through HIV service delivery and outreach settings facilitated by partnering nongovernmental organizations, supplemented by online LGBTQ+ (lesbian, gay, bisexual, transgender, queer, and other sexual and gender minorities) networks. The study used nonprobability convenience sampling with prespecified eligibility criteria. Data collection was conducted from January 2025 to May 2025. The sample comprised 141 platform users and 27 health care providers (N=168). An extended UTAUT model incorporated performance expectancy (PE), effort expectancy (EE), social influence (SI), facilitating conditions (FC), trust, and perceived stigma (PS). Data were analyzed using correlation analysis and ordinary least squares (OLS) regression. Age and educational attainment were examined as potential moderating variables. Qualitative feedback was also collected to contextualize quantitative findings.

**Results:**

FC emerged as the strongest predictor of BI to use the platform (β=.813; *P*<.001), explaining a substantial proportion of variance in adoption intention (*R*²=0.652). Age demonstrated a modest but statistically significant positive effect on BI (β=.105; *P*=.03). Contrary to theoretical expectations, PE, EE, SI, trust, and PS did not significantly predict intention to adopt the platform. Qualitative feedback highlighted practical infrastructure-related concerns, including reliable internet access, offline functionality, and availability of technical support, as more salient than psychological or feature-based considerations.

**Conclusions:**

In resource-constrained, highly stigmatized contexts, enabling infrastructure and practical support may outweigh cognitive, social, and attitudinal determinants of technology acceptance. Successful digital health interventions for marginalized populations therefore require privacy-conscious, user-centered design alongside sustained investment in the infrastructure that supports real-world use.

## Introduction

Despite decades of progress in HIV treatment, sub-Saharan Africa continues to shoulder a disproportionate burden of infection and mortality [1]. Within this region, men who have sex with men (MSM) and transgender people are particularly vulnerable due to intersecting clinical, social, and legal factors. They experience higher HIV prevalence, reduced access to prevention and treatment services, and heightened exposure to stigma, discrimination, and violence.

Digitalization is reshaping how health services are designed and delivered, including the expansion of remote consultation, mobile health (mHealth) apps, and digitally mediated access to medicines and counseling. In HIV care, digital pathways can be particularly valuable where individuals face practical barriers (distance, time, and cost) and social barriers (fear of disclosure, stigma, and discrimination). These barriers are often intensified for MSM and transgender people in contexts where sexual and gender minorities experience marginalization, safety concerns, and reduced willingness to engage with clinic-based services. Although digital interventions promise improved continuity of care and confidentiality, adoption is not automatic; it depends on perceived value, usability, social context, enabling conditions, and trust in how sensitive information is handled [2-4]. A systematic review and meta-analysis of counseling support for HIV self-testing among MSM similarly underscores the centrality of structured counseling and linkage-to-care pathways within digital HIV interventions, reinforcing the case for embedding counseling and education functions, not only consultation scheduling, within platforms such as TechAids, developed by EO (lead author) [5].

Digital health technologies, including mHealth apps, teleconsultations, and online medication delivery, have been proposed as a way to extend care beyond physical clinic walls and offer discreet, flexible access to services. Evidence suggests that mHealth interventions can improve adherence to treatment and support self-management in chronic conditions [6], and that digital platforms can increase access to care in underserved populations [7]. However, acceptance of such tools depends on a complex interplay of perceived usefulness, usability, trust, privacy, and the structural conditions that enable or constrain use [8,9]. A recent randomized controlled trial of a chatbot-delivered HIV self-testing support intervention among MSM found it to be noninferior to human-delivered real-time support in increasing self-testing uptake and linkage to counseling, illustrating that digitally mediated, low-cost support models can match human-delivered care for key populations in other global settings [10].

The Unified Theory of Acceptance and Use of Technology (UTAUT), developed by Venkatesh et al [2], provides a well-established framework for understanding why individuals adopt or reject information systems. UTAUT has been widely applied across health care contexts, from electronic health record adoption by clinicians [11] to patient portal usage [12] and mHealth applications [13]. However, standard UTAUT constructs may inadequately capture the psychological and social complexities of HIV care, particularly for criminalized populations. HIV-related stigma, both anticipated and enacted, profoundly shapes health care-seeking behavior [14]. For MSM and transgender individuals, this stigma operates multiplicatively, intersecting with sexual orientation and gender identity stigma to create what researchers term “layered” or “syndemic” stigma [15,16].

Similarly, trust in health technologies becomes paramount when sharing sensitive information about HIV status, sexual behavior, and gender identity could expose individuals to discrimination or legal jeopardy [17,18]. McKnight et al [19] conceptualized trust in technology as comprising beliefs about reliability, functionality, and helpfulness, alongside faith in the provider’s benevolence and integrity. In stigmatized health domains, these dimensions acquire heightened salience; users must trust not only that systems will function reliably, but that data will be protected and that no harm will result from engagement.

This study examined BI to adopt TechAids, a digital platform developed specifically for discreet HIV service access in Nigeria. TechAids provides teleconsultation scheduling, medication delivery coordination, appointment reminders, and educational resources through a mobile app. The platform emphasizes privacy through end-to-end encryption, anonymous registration options, and discreet packaging for medication delivery. We extended the UTAUT framework by incorporating trust and Perceived Stigma (PS) as additional predictors, recognizing that standard technology acceptance models may inadequately represent the lived realities of marginalized populations navigating highly stigmatized health conditions.

Our research addressed three primary questions:

1. Which factors most strongly predict intention to adopt TechAids among MSM and transgender individuals living with HIV in Nigeria?
2. Do trust and PS play significant roles beyond standard UTAUT constructs in predicting adoption intention?
3. Do demographic characteristics, specifically age and education, moderate the relationships between UTAUT constructs and BI, suggesting the need for differentiated implementation strategies across user segments?

## Methods

### Theoretical Framework and Hypotheses

We built our conceptual model on UTAUT’s foundation while extending it to address the unique considerations of digital health platforms serving stigmatized populations. The UTAUT establishes a comprehensive framework that helps understand how users accept or reject technology solutions and how this shapes their behavioral patterns. In this work, we used the original UTAUT baseline model from Venkatesh et al [2]. This is taken up and elaborated in the context of a stigma-aware digital health context.

Performance expectancy (PE) represents the degree to which individuals believe that using TechAids will help them manage their HIV care more effectively [2]. In this context, PE encompasses beliefs about whether the platform will improve access to medications, simplify appointment scheduling, enhance communication with health care providers, and ultimately contribute to better health outcomes. Prior research in HIV mHealth suggests that perceived utility for medication adherence and symptom monitoring strongly predicts app usage [18].

Effort expectancy (EE) captures perceived ease of use—the extent to which potential users anticipate that interacting with TechAids will be free from difficulty [2]. For populations with varying levels of digital literacy, interface simplicity and intuitive navigation become critical adoption factors [20]. Given that many MSM and transgender individuals in Nigeria access smartphones but may have limited experience with health applications, EE could substantially influence adoption intentions.

Social influence (SI) reflects the degree to which important others’ opinions affect technology adoption decisions [2]. Within tight-knit MSM and transgender communities, peer recommendations and community advocate endorsements may carry particular weight [21]. However, social influence operates ambivalently in stigmatized contexts, while peer support may encourage adoption, concerns about community gossip or inadvertent disclosure might inhibit it [17].

Facilitating conditions (FC) encompasses perceptions of available resources and infrastructure support for platform use [2]. This construct includes access to smartphones and reliable internet connectivity, compatibility with existing devices, availability of technical assistance, and adequacy of bandwidth for app functionality. In sub-Saharan African contexts, where internet penetration remains uneven and data costs consume significant portions of household budgets, FC frequently emerge as critical determinants of mHealth adoption [22].

Trust represents confidence that TechAids will reliably protect sensitive information and operate in users’ best interests [19]. We conceptualized trust as comprising 3 dimensions: confidence in the platform’s technical security (data encryption and secure storage), faith in the organization’s benevolence (commitment to user welfare), and belief in institutional integrity (honest representation of capabilities and limitations). For platforms handling HIV-related data, trust becomes foundational; users must believe their information will not be compromised, sold, or disclosed to authorities [17].

Perceived stigma (PS) captures individuals’ concerns about potential discrimination, judgment, or social consequences resulting from platform use. We measured both anticipated stigma (expectations of being devalued if HIV status becomes known) and internalized stigma (negative self-perceptions related to HIV). While TechAids explicitly addresses stigma through confidentiality features, PS might still deter adoption if users worry about visible app icons, delivery package identification, or data breaches.

We also examined age and education as potential moderators, recognizing that technology adoption patterns often vary across demographic groups [2]. Younger individuals typically demonstrate greater digital fluency, while education level may influence both technical competence and health care information processing.

Education and counseling are also core, rather than peripheral components of HIV care, and this is reflected directly in TechAids’ design: alongside teleconsultation and medication delivery, the platform provides educational resources intended to support adherence counseling and risk-reduction communication. We therefore situate PE and FC in this study not only as technology-acceptance constructs but also as proxies for whether the platform’s behavioral and counseling functions are perceived as effective and accessible. This framing is consistent with the evidence that structured counseling support substantially improves linkage to care following HIV self-testing among MSM [5], reinforcing the theoretical rationale for treating educational and counseling content as integral to, rather than separate from, the technology-acceptance constructs examined here.

Figure 1 illustrates the hypothesized relationships among constructs.

**Figure 1.**
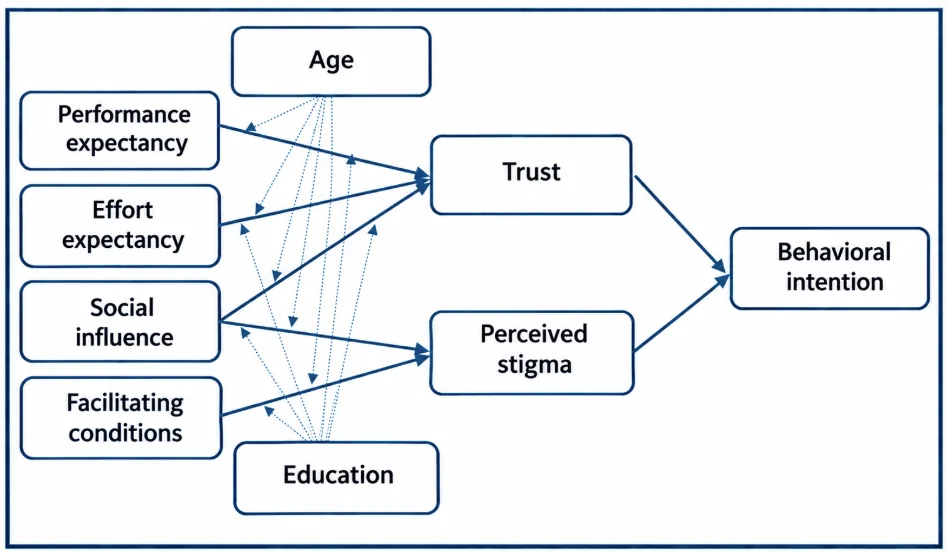
Extended UTAUT model for TechAids adoption. Source: adapted from Venkatesh et al [2]. UTAUT: Unified Theory of Acceptance and Use of Technology.

### Research Hypotheses

Research hypotheses serve as a cornerstone of empirical research. They provide a focused framework for data collection and analysis and are essential for confirming or refuting theoretical assumptions. Sekaran and Bougie [23] define a research hypothesis as a logically conjectured relationship between 2 or more variables expressed in the form of a testable statement. Developing research hypotheses through UTAUT enables examination of the theoretical connections between different constructs. They establish the direction for data collection and analysis while enabling researchers to validate or adjust the UTAUT model through empirical evidence in various contexts. Based on the specific context of TechAids, Multimedia Appendix 1 summarizes the hypotheses.

### Study Design and Setting

This study adopted an explanatory mixed methods design combining quantitative survey data assessing UTAUT constructs with qualitative open-ended responses about platform preferences and concerns. The quantitative component tested an extended UTAUT model using ordinary least squares (OLS) models while the qualitative component used thematic analysis of open-ended responses to triangulate statistical findings and provide contextual interpretation. The UTAUT model created by [2] was used in the study to identify the variables influencing MSM and transgender individuals’ acceptance and use of the TechAids app. According to the literature, the UTAUT model, which is used in various communities and research fields, can benefit from varying scale items and sizes. Items were organized into 7 latent constructs aligned with an extended UTAUT framework: PE, EE, SI, FC, trust, PS, and BI, alongside age and education. Constructs were operationalized using multiitem scales adapted from prior technology acceptance and e-health research. All items were rated on a 5-point Likert scale (1=strongly disagree to 5=strongly agree). Explanation was made to discuss the use of the scale to ensure a clear understanding of the items by the researchers before completing the survey.

### The TechAids Platform

TechAids is a mobile-optimized digital platform developed through participatory design workshops with Nigerian MSM and transgender communities [24]. The platform offers 4 core functions: (1) teleconsultation scheduling with trained HIV health worker clinicians, (2) confidential medication delivery coordination with discreet packaging, (3) automated appointment and medication reminders, (4) educational resources about HIV treatment and wellness. Security features include end-to-end encryption for all communications and automatic session timeouts. For this study, we developed a functional prototype incorporating these features to allow participants hands-on exploration before completing surveys.

### Participants and Recruitment

We used nonprobability convenience sampling in recruiting participants who were accessible through HIV clinics and community service settings facilitated by The Initiative for Equal Rights (TIERs) and the Happiest Ones Health Support & Rights Initiative (HOHSRI), together with online LGBTQ+ (lesbian, gay, bisexual, transgender, queer, and other sexual and gender minorities) networks in Lagos, Nigeria. Eligibility criteria for the user group included (1) age 18 years or older, (2) self-identification as MSM, transgender, or gender diverse, (3) confirmed HIV-positive status or high engagement in HIV prevention services, (4) ownership of or regular access to a smartphone, and (5) ability to provide informed consent. Recruitment materials emphasized voluntary participation, assured confidentiality, and clarified that declining to participate would not affect access to any health services. Nongovernmental organizations (NGOs) serve as gatekeepers for the recruitment of participants to build trust and minimize coercion concerns.

Criteria (2) and (3) were applied disjunctively ie, participants qualified by self-identifying as MSM, transgender, or gender diverse, or by having confirmed HIV-positive status or high engagement in HIV prevention services. This explains why, as reported in Multimedia Appendix 2, most participants identified as heterosexual and HIV-negative. Health care providers were recruited separately through the same NGOs and were eligible if they currently delivered HIV-related services to MSM or transgender clients. Providers and users were analyzed within a single regression model because both were prospective adopters of the same platform and UTAUT items were worded to apply across both roles. Recruitment proceeded via NGO and clinic staff introductions during routine service contact or outreach events, supplemented by online LGBTQ+ network recruitment. Recruitment source was not recorded, precluding a breakdown by channel. This was noted as a limitation of recruitment tracking.

Data collection and survey instrument: Participants interacted with the TechAids functional prototype and then completed a structured questionnaire comprising 28 Likert-scale items covering PE, EE, SI, FC, trust, PS, and BI. The measurement items are presented in Multimedia Appendix 3. The questionnaire also included 3 open-ended questions: (1) What are the things you like about a mobile app for consultation and medication delivery? (2) What are the things you dislike about using a mobile app for consultation and medication delivery? and (3) What do you think would make TechAids App more useful for people living with HIV and AIDS? A cross-sectional survey was conducted from January 2025 to May 2025.

### Ethical Considerations

The Bournemouth University Research Ethics Committee approved this study (Ethics ID 51057). Given the sensitivity of research involving criminalized populations and HIV status, we implemented enhanced confidentiality protections. All data were stored on password-protected servers without personal identifiers and transferred via secure protocols.

Informed consent was obtained from all participants prior to enrollment; consent procedures explained the study’s purpose, the voluntary nature of participation, the right to withdraw at any time without affecting access to health services, and the confidentiality protections in place, and were delivered verbally or in writing according to participant preference and literacy. To mitigate risk associated with participation in a criminalized legal context, study staff member (EOO) avoided collecting or recording any information that could independently identify a participant’s sexual orientation, gender identity, or HIV status outside the secure survey instrument; prototype testing sessions were conducted in private spaces arranged by partnering NGOs to minimize visibility to others; and participants were not required to disclose sensitive identity or status items, consistent with the “prefer not to say” response option used throughout the survey. Data linking participant identities to survey responses were not retained beyond the minimum required for recruitment logistics, and all analytic data were fully deidentified prior to analysis.

### Data Analysis

A priori power analysis using G*Power 3.1 (developed by Franz Faul) [25] was used to determine the minimum sample size. We conducted analyses using Statistical Package for the Social Sciences (SPSS) version 28 (IBM Corp.) [26].

### Estimation Approach

OLS regression was used to estimate the relationships between the independent variables and BI to use the digital health platform. The operationalization of each theoretical variable was based on a composite index which consisted in averaging the responses for their corresponding Likert-scale indicators from validated measurement scales. This procedure allowed the constructs to be treated as continuous observed values that are suitable for regression analysis. The reliabilities of the scales were evaluated by Cronbach α during model estimation to examine internal consistency between the components [27]. Descriptive statistics and a Pearson correlation analysis were carried out to examine distributions of variables and preliminary associations.

Treating averaged multiitem Likert composites as approximately continuous for OLS estimation is a widely used convention in technology-acceptance survey research, including prior UTAUT studies in similar low-resource settings [28], and is generally considered acceptable when each composite is derived from four or more reliable indicators, as is the case for all constructs here (Multimedia Appendix 3). BI, like the other UTAUT constructs, reflects an underlying latent variable rather than a strictly interval-level measure, and that ordinal regression or structural equation modeling (SEM) would provide a valuable robustness check on the OLS findings reported below, particularly given the unusually strong association observed between FC and BI. This is a methodological limitation and direction for future analysis.

Composite reliability (CR) = $\frac{{\left(\sum \lambda_{i}\right)}^{2}}{{\left(\sum \lambda_{i}\right)}^{2}+\sum \left(1-\lambda_{i}^{2}\right)}$where (lambda) is the standardized factor loading.

Average variance extracted (AVE) = $\frac{\sum \lambda_{i}^{2}}{n}$, where n is the number of indicators per construct

Predictive accuracy was evaluated through the coefficient of determination (*R*^2^), which represents the proportion of variance in BI explained by the predictor variables. The OLS regression equation was estimated using standardized coefficients to facilitate comparison of effect sizes across predictors.


$${\text{R}}_{\text{BI}}^{2}=1-\frac{\sum {\left({\text{BI}}_{\text{obs}}-{\text{BI}}_{\text{pred}}\right)}^{2}}{\sum {\left({\text{BI}}_{\text{obs}}-{\overset{-}{\text{BI}}}_{\text{obs}}\right)}^{2}}$$

A priori power analysis conducted using G*Power (effect size *f*^2^=0.15, significance level *α*=.05, statistical power=0.80, and 8 predictors) indicated a minimum required sample size of 109. The final sample size of 168 therefore exceeded this threshold, confirming adequate statistical power for detecting medium-sized effects.

### Measurement of Reliability and Validity of UTAUT Constructs

Reliability analysis was conducted to assess the internal consistency of the items for each latent factor of the UTAUT model before going into the model testing. Cronbach α was applied to ensure that the scale used for rating (Likert scale) is reliable. Multimedia Appendix 3 shows the Cronbach α scores achieved for each factor. An acceptable minimum threshold for Cronbach α is suggested to be 0.7 according to [29,30].

### Relationships Among Constructs

#### Correlation Analysis

The study used correlation analysis to evaluate the strength and direction of the linear relationship between 2 variables. Correlation analysis helps to determine which factors are most strongly associated with users’ willingness to adopt and use health technologies. A positive correlation indicates that as 1 variable increases (ie, perceived usefulness), the other (ie, intention to use the app) also increases. The coefficient (ρ) value ranges from −1 to +1. Quantitative analysis techniques like correlation are essential for testing the relationships among these constructs. The Pearson correlation coefficient is used to examine the strength and direction of linear relationships between UTAUT constructs ie,


$$\rho_{XY}=\frac{\sum (X_{i}-\bar{X})\sum (Y_{i}-\bar{Y})}{\sqrt{\sum (X_{i}-\bar{X}{)}^{2}\sum (Y_{i}-\bar{Y}{)}^{2}}}$$

where X_i_= scores on construct X (eg, PE); Y_i_= scores on construct Y (eg, BI); $\bar{\text{X}}$ and $\bar{\text{Y}}$ are means of X and Y respectively.

#### Regression Analysis

OLS regression was used to help determine which constructs significantly impact BI to use the technology. It provides insights into which factors are most influential to enhance technology adoption. Regression analysis predicts the value of a dependent variable (eg, BI) based on independent variables (eg, PE, EE, SI, FC, trust, and PS). We estimated a set of standardized OLS regression models that map directly onto the extended UTAUT structure.

The baseline regression model is specified as follows:


$$\begin{array}{rl} BI & =\beta_{0}+\beta_{1}PE_{i}+\beta_{2}EE_{i}+\beta_{3}SI_{i}+\beta_{4}FC_{i}\\ \;+\beta_{5}TR_{i}+\beta_{6}PS_{i}+\beta_{7}Age_{i}\\ \;+\beta_{8}Edu_{i}+\varepsilon_{i} \end{array}$$

Each construct is a latent variable represented by a set of observed indicators. For example,


$$\begin{array}{rl} PE_{i} & =\lambda_{PE1}X_{PE1,i}+\lambda_{PE2}X_{PE2,i}+\lambda_{PE3}X_{PE3,i}\\ \;+\lambda_{PE4}X_{PE4,i}+\delta_{PE,i} \end{array}$$

$$\begin{array}{rl} EE_{i} & =\lambda_{EE1}X_{EE1,i}+\lambda_{EE2}X_{EE2,i}+\lambda_{EE3}X_{EE3,i}\\ \;+\lambda_{EE4}X_{EE4,i}+\delta_{EE,i} \end{array}$$

$$SI_{i}=\lambda_{SI1}X_{SI1,i}+\lambda_{SI2}X_{SI2,i}+\lambda_{SI3}X_{SI3,i}+\lambda_{SI4}X_{SI4,i}+\delta_{SI,i}$$

$$\begin{array}{rl} FC_{i} & =\lambda_{FC1}X_{FC1,i}+\lambda_{FC2}X_{FC2,i}+\lambda_{FC3}X_{FC3,i}\\ \;+\lambda_{FC4}X_{FC4,i}+\delta_{FC,i} \end{array}$$

$$\begin{array}{rl} TR_{i} & =\lambda_{TR1}X_{TR1,i}+\lambda_{TR2}X_{TR2,i}+\lambda_{TR3}X_{TR3,i}\\ \;+\lambda_{TR4}X_{TR4,i}+\delta_{TR,i} \end{array}$$

$$\begin{array}{rl} PS_{i} & =\lambda_{PS1}X_{PS1,i}+\lambda_{PS2}X_{PS2,i}+\lambda_{PS3}X_{PS3,i}\\ \;+\lambda_{PS4}X_{PS4,i}+\delta_{PS,i} \end{array}$$

$$\begin{array}{rl} Age_{i} & =\lambda_{Age1}X_{Age1,i}+\lambda_{Age2}X_{Age2,i}+\lambda_{Age3}X_{Age3,i}\\ \;+\lambda_{Age4}X_{Age4,i}+\delta_{PS,i} \end{array}$$

$$\begin{array}{rl} Edu_{i} & =\lambda_{PS1}X_{PS1,i}+\lambda_{PS2}X_{PS2,i}+\lambda_{PS3}X_{PS3,i}\\ \;+\lambda_{PS4}X_{PS4,i}+\delta_{PS,i} \end{array}$$

where $\lambda_{PE_{i}},\;\lambda_{EE_{i}},\;\lambda_{SI_{i}},\;\lambda_{FC_{i}},\;\lambda_{TR_{i}},\;\lambda_{PS_{i}}$, are factor loadings, $X_{PEi},\;X_{EEi},\;X_{SIi},\;X_{FCi},\;X_{TRi},\;X_{PSi}$, are measured items, and $\delta_{PE},\;\delta_{EE},\;\delta_{SI},\;\delta_{FC},\;\delta_{TR},\;\delta_{PS}$ represent measurement errors.

#### Moderation Analysis

To test moderation hypotheses H7 and H8, interaction terms were created between each UTAUT construct and both demographic moderators (age and education). Both moderators were mean-centered before creating product terms to reduce multicollinearity. Eight separate regression models were then estimated, each including the full set of main effects plus one interaction term, consistent with the estimation approach described above.

#### Categorization of Qualitative Data

Open-ended responses to the 3 qualitative survey questions (Table 1) were analyzed using an inductive, data-driven thematic-categorization approach, following the reflexive thematic analysis approach described by Braun and Clarke [31]. Analysis proceeded in 4 steps: (1) the lead author (EOO) read all open-ended responses in full to gain familiarity with the data; (2) initial descriptive labels were generated inductively from the responses, question by question, rather than applied from a preexisting framework; (3) labels were iteratively grouped and refined into a smaller set of categories per question as additional responses were reviewed with earlier responses rechecked against revised category definitions; and (4) the resulting categories were tabulated against illustrative response counts (Table 1). Coding was conducted by a single coder, EOO (the lead author); no second coder independently coded the data, and consequently, no formal interrater reliability statistic (eg, Cohen kappa) was calculated and no coding discrepancies required resolution through discussion or arbitration. The categorization should therefore be read as a descriptive thematic summary intended to contextualize the quantitative findings, rather than as output from an independently verified, consensus-based qualitative analysis. Independent double-coding, with formal interrater reliability assessment, is identified as a priority for future mixed methods work with this dataset. This is revisited in the Limitations section.

**Table 1.** Qualitative feedback from the survey.

Serial number	Survey questions	Categorization of the survey responses
1.	What are the things you like about a mobile app for consultation and medication delivery?	Access and convenience. Personalization and control. Privacy and security. Medication management and delivery. Communication and support. Health information and education.
2.	What are the things you dislike about using a mobile app for consultation and medication delivery?	User experience. Technical performance and app stability. Access to specialists and services. Internet dependence and connectivity.
3.	What do you think would make TechAids app more useful for people living with HIV or AIDS?	Access to educational content Access to health care providers End-to-end encryption Free and fast internet Offline functionality Quick helpline access Regular app update Smart reminder

### Research Participants

The study population of this research consists of 168 respondents who participated in the completion of a survey which consisted of 2 stakeholders ie, health care providers and patients. The respondents that did not complete the survey questions were expunged from the sample. On average, completion of the survey took approximately 32 minutes, which could contribute to the reason some respondents did not complete the survey. The survey focuses on evaluating the willingness to adopt the use of digital health care technologies among MSM and transgender individuals living with HIV/AIDS. The relatively small sample size in this study reflects practical constraints associated with conducting research in underserved communities [32]. The research participants were given some tasks to perform on the TechAids app’s prototype. The tasks completed are highlighted in Figure 2 below.

**Figure 2.**
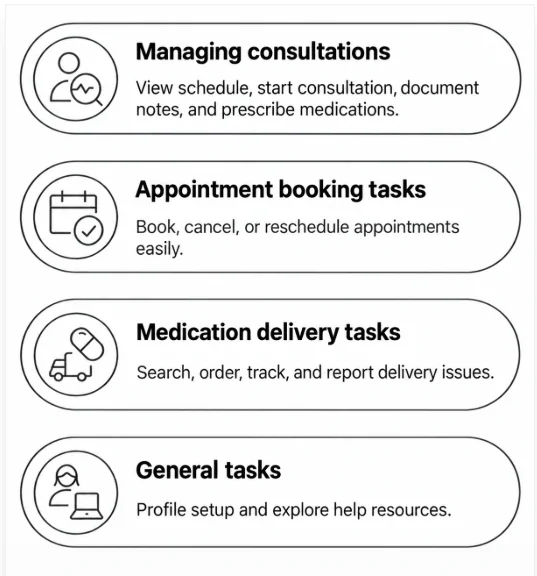
Prototype design tasks.

## Results

### Overview

Two sets of results from the UTAUT-based survey were presented in this section. First, quantitative analysis, and especially descriptive and inferential analyses, were used to explore key factors influencing the use and acceptance of technology. Second, the qualitative data were analyzed using a categorization of ideas approach, highlighting themes and patterns in participants’ perceptions and experiences. Both qualitative and quantitative methods offer a comprehensive understanding of technology adoption in HIV/AIDS care among MSM and transgender people living with HIV/AIDS.

### Participant Characteristics

Multimedia Appendix 2 summarizes demographic characteristics. The final sample comprised 141 platform users (patients) and 27 health care providers (n=168). User participants averaged 31.2 (SD 4.7, range 21‐40) years. Nearly half (n=69, 48.9%) fell in the 31‐ to 40-year age bracket, while 51.1% (n=72) were aged 21‐30 years. Educational attainment was relatively high: 34% (48/141) held master’s degrees, 30.5% (43/141) bachelor’s degrees, and 35.5% (50/141) diplomas or equivalent technical qualifications.

Gender identity patterns reflected the sensitivity of these questions in the Nigerian context. Among users, 31.2% (44/141) identified as male, 36.2% (51/141) as female, and 32.6% (46/141) preferred not to disclose. Only 2 participants (2/141, 1.4%) explicitly identified as transgender, though an additional 46.8% (66/141) preferred not to answer this question, suggesting possible transgender identity among nondisclosers. Nearly half (69/141, 48.9%) declined to state sexual orientation, while 49.6% (70/141) identified as heterosexual and 1.4% (2/141) as gay or lesbian. These patterns likely reflect justified caution given Nigeria’s criminalization of same-sex relationships.

Regarding HIV status, 54.6% (77/141) of users indicated HIV-negative status (likely reflecting engagement in prevention services), while 45.4% (64/141) declined to disclose, a pattern consistent with pervasive HIV stigma. Health care provider participants (n=27) averaged slightly older (19/27, 70.4% aged 31‐40 years) and showed similar reluctance to disclose personal information on sensitive items.

### Reliability Analysis

Consistency of the constructs of the model was measured using Cronbach α. Internal consistency was assessed for each multiitem construct, targeting α*≥*0.70. Construct validity was evaluated using exploratory factor-analytic logic, together with the interconstruct correlation matrix among the 7 construct means. Factor analysis was used to investigate underlying relationships (called factors) among a set of observed variables. It helps reduce a large number of variables into fewer unobserved variables (factors) that explain the patterns in the data. This was used to generate the factor loadings for the predictors.

All constructs demonstrated acceptable to excellent internal consistency (Multimedia Appendix 3). Cronbach α values ranged from 0.701 (BI) to 0.887 (trust), well exceeding the 0.70 threshold [27]. Trust showed the highest reliability, suggesting that items measuring confidence, security, and confidentiality concerns cohered strongly. Item-total correlations within each construct ranged from 0.52 to 0.84, indicating that individual items contributed meaningfully to their respective scales. Factor loadings from exploratory factor analysis ranged from 0.668 to 0.903, confirming that items loaded strongly on their intended constructs. CR and AVE computed from single-factor loadings extracted per construct via principal axis factoring are reported alongside Cronbach α in Multimedia Appendix 3. All 7 constructs exceeded the conventional CR threshold of 0.70 (range: 0.707 to 0.889). AVE exceeded the conventional 0.50 threshold for 5 of the 7 constructs (SI=0.509, FC=0.561, trust=0.669, PS=0.572), while PE (AVE=0.429), EE (AVE=0.457), and BI (AVE=0.382) fell modestly below this threshold, indicating that convergent validity for these 3 constructs, while supported by adequate CR and Cronbach α was not fully confirmed by AVE alone. This was reported transparently as a measurement limitation in the Limitation and Future Research section rather than treating CR or alpha as sufficient on their own. A Fornell-Larcker discriminant-validity check, comparing the square root of each construct’s AVE against its correlations with all other constructs (Table 2), confirmed discriminant validity for all construct pairs except FC and BI: the FC-BI correlation (*r*=0.802) exceeded both constructs’ √AVE (0.749 and 0.618, respectively), indicating a discriminant-validity concern between these 2 constructs specifically. This finding statistically corroborates, rather than merely conceptually motivates, the caution urged elsewhere in this study regarding the magnitude of the FC–BI association and is consistent with the possibility of conceptual or item-level overlap between these 2 constructs that should be addressed through item refinement in future research. A full confirmatory factor analysis was not conducted, given sample size constraints relative to the number of constructs and items; this is noted as a limitation.

**Table 2.** Correlation matrix showing relationships between UTAUT^a^ constructs and ^b^BI to use the Techaids app. The extremely high ^c^FC-BI^b^ correlation (*r*=0.802) raised concerns about multicollinearity that we addressed through VI^d^F analysis in regression models.

Constructs	Age	Education	PE^e^	EE^f^	SI^g^	FC^c^	Trust	PS^h^	BI^b^
Age	1	−0.115	−0.035	0.012	0.058	−0.065	0.014	0.087	0.048
Education	−0.115	1	0.013	−0.105	−0.154	−0.016	0.068	−0.027	−0.018
PE	−0.035	0.013	1	−0.033	−0.152	0.004	0.003	0.047	−0.071
EE	0.012	−0.105	−0.033	1	0.092	0.032	0.038	0.203	0.038
SI	0.058	−0.154	−0.152	0.092	1	0.048	0.067	0.028	0.025
FC	−0.065	−0.016	0.004	0.032	0.048	1	−0.088	0.059	0.802
Trust	0.014	0.068	0.003	0.038	0.067	−0.088	1	−0.011	−0.078
PS	0.087	−0.027	0.047	0.203	0.028	0.059	−0.011	1	0.003
BI	0.048	−0.018	−0.071	0.038	0.025	0.802	−0.078	0.003	1

a UTAUT: Unified Theory of Acceptance and Use of Technology.

b BI: behavioral intention.

c FC: facilitating conditions.

d VIF: variance inflation factor.

e PE: performance expectancy.

f EE: effort expectancy.

g SI: social influence.

h PS: perceived stigma.

### Correlation Analysis

Hypotheses were tested using correlation coefficient. This was used to find the linear relationship between 2 constructs. Table 2 shows the correlation coefficients between multiple variables. Each cell in the matrix reflects the strength and direction of the linear relationship between 2 variables using a value between −1 and +1.

Table 2 presents zero-order correlations among all study variables. FC showed a remarkably strong positive correlation with BI (*r*=0.802; *P*<.001), a magnitude rarely observed in technology acceptance research. This relationship was notably stronger than correlations between other UTAUT constructs and intention: PE (*r*=−0.071; *P*=.37), EE (*r*=0.038; *P*=.63), and SI (*r*=0.025; *P*=.75) showed negligible associations with intention. Trust correlated negatively with intention (*r*=−0.078; *P*=.32), while PS showed essentially zero correlation (*r*=0.003; *P*=.97).

Age demonstrated weak positive correlations with BI (*r*=0.048; *P*=.54) and PS (*r*=0.087; *P*=.26). Education showed small negative correlations with most constructs, but none reached statistical significance. The intercorrelations among UTAUT predictors were generally weak, with 1 notable exception: EE and PS correlated at *r*=0.203 (*P*=.008), suggesting that individuals who anticipated greater stigma also perceived the platform as more difficult to use.

### Regression Analysis

The relationship between the independent variables X (ie, PE, EE, SI, FC, TR, PS, age, and education) and the dependent variable Y (BI) are shown in Table 3. Each coefficient quantifies how much the dependent variable is expected to increase or decrease when the corresponding independent variable increases by one unit, assuming all other variables are held constant.

Table 3 presents regression results predicting BI. The overall model explained substantial variance: *R*²=0.652, *F*_8, 159_=37.25; *P*<.001, indicating that our extended UTAUT model accounted for approximately 65% of variation in adoption intentions. This model was estimated on the combined sample of platform users and health care providers (n=168), as justified in Participants and Recruitment section. As a sensitivity check, we reestimated the model restricted to the (n=141) platform users only. The pattern of results closely replicated the combined-sample findings: FC remained the dominant predictor and, if anything, showed a slightly larger standardized effect in the user-only model (β=.856, *t*_132_=19.37; *P*<.001) than in the combined sample (β=.813), and age remained a significant, modestly sized positive predictor (β=.133; *P*=.003 in the user-only model vs β=.105; *P*=.03 in the combined sample). PE, EE, SI, trust, PS, and education remained nonsignificant in both models, and the user-only model explained more variance overall (*R*²=0.746 vs *R*²=0.652 for the combined sample). The significance pattern across all 8 predictors was therefore identical between the combined and user-only samples (Table 4), indicating that including health care providers in the primary analysis did not materially distort the substantive conclusions of this study.

**Table 3.** Regression analysis results for UTAUT^a^ constructs predicting Techaids app usage intention.

Constructs	Unstandardized β	Standardized β	SE (β)	*t* test (*df*)	Significance level	VIF^b^
Intercept	1.446	0	0.482	2.99 (159)	.003	—^c^
PE^d^	−0.081	−.071	0.053	−1.53 (159)	.13	1.029
EE^e^	0.024	.023	0.050	0.48 (159)	.63	1.064
SI^f^	−0.032	−.030	0.050	−0.63 (159)	.53	1.065
FC^g^	0.720	.813^h^	0.041	17.47^h^ (159)	<.001^h^	1.020^h^
Trust	−0.007	−.008	0.043	−0.16 (159)	.87	1.022
PS^i^	−0.056	−.055	0.049	1.156 (159)	.25	1.059
Age	0.014	.105^h^	0.006	2.253^h^ (159)	.03^h^	1.030^h^
Education	0.003	.005	0.023	0.108 (159)	.91	1.053

a UTAUT: Unified Theory of Acceptance and Use of Technology.

b VIF: variance inflation factor.

c Not applicable.

d PE: performance expectancy.

e EE: effort expectancy.

f SI: social influence.

g FC: facilitating conditions.

h *P*<.05.

i PS: perceived stigma.

**Table 4.** Comparison of standardized regression coefficients, combined sample (n=168) vs platform-users-only subsample (n=141). *R*²=0.652 (combined) vs 0.746 (users-only).

Predictor	Combined β (n=168)	*P* values	Users-only β (n=141)	*P* values
PE^a^	−.071	.13	−.038	.40
EE^b^	.023	.63	.035	.45
SI^c^	−.030	.53	−.027	.55
FC^d^	.813	<.001	.856	<.001
Trust	−.008	.87	−.026	.56
PS^e^	−.055	.25	−.051	.27
Age	.105	.03	.133	.003
Education	.005	.91	−.039	.39

a PE: performance expectancy.

b EE: effort expectancy.

c SI: social influence.

d FC: facilitating conditions.

e PS: perceived stigma.

In the regression analysis, FC emerged as the overwhelmingly dominant predictor (β=.813; SE=.041; t_159_=17.47; *P*<.001) (Figure 3). The standardized coefficient magnitude indicates that a one-standard-deviation increase in perceived FC corresponded to a .720 standard deviation increase in intention to adopt TechAids—an exceptionally large effect. Age showed a small but statistically significant positive effect (β=.105, SE=.006, *t*_159_=2.253; *P*=.03), suggesting that older participants within our age range expressed slightly greater adoption intention.

Contrary to our hypotheses, PE (β=−.071; *P*=.13), EE (β=.023; *P*=.63), SI (β=−.030; *P*=.53), trust (β=−.008; *P*=.87), and PS (β=−.055; *P*=.25) did not significantly predict BI. Education also showed no effect (β=.005; *P*=.91).

**Figure 3.**
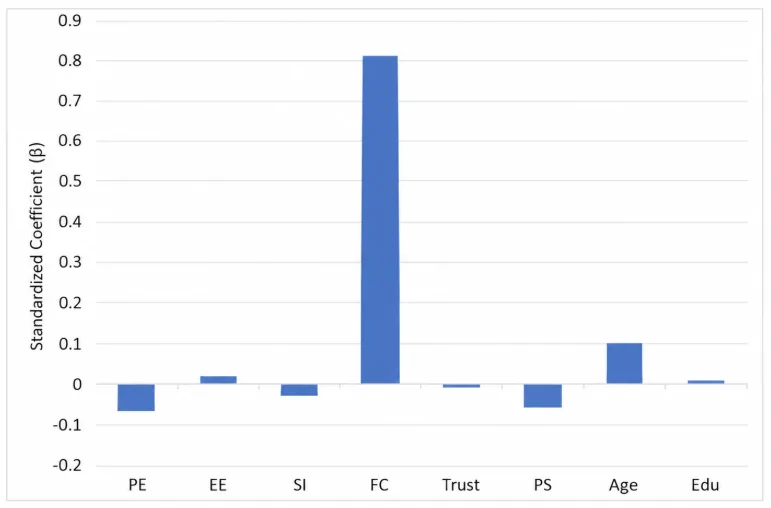
Standardized beta (β) of UTAUT constructs predicting intention to use the TechAids App. EE: effort expectancy; FC: facilitating conditions; PE: performance expectancy; PS: perceived stigma; SI: social influence; UTAUT: Unified Theory of Acceptance and Use of Technology.

We examined variance inflation factors (VIFs) to assess multicollinearity, which indicated no cause for concern. FC showed VIF=1.02, well below conventional thresholds of 5, indicating that its strong effect was not attributable to collinearity with other predictors. All other VIF values ranged from 1.022 to 1.065 (Table 3), suggesting minimal shared variance among predictors (independent variables). Tolerance values, the reciprocal of VIF, confirmed this pattern (range 0.939‐0.980) with all values well above the conventional concern threshold of 0.10. The Durbin-Watson statistic was 1.782, within the conventionally acceptable range of 1.5 to 2.5, indicating no evidence of autocorrelation among residuals. Case-level influence diagnostics likewise raised no concerns: the Cook distance ranged from approximately 0 to 0.140 (mean 0.006, SD 0.014) with no case exceeding the threshold of 1.0 conventionally used to flag seriously influential observations; 8 cases (4.8%) exceeded the more conservative screening threshold of 4 /n, a proportion consistent with chance variation at this sample size. Leverage (hat) values ranged up to 0.192 (mean 0.054, SD=0.026), with 8 cases (4.8%) exceeding the 2(k+1)/n screening threshold; critically, no case was flagged by both the Cook distance and leverage simultaneously, indicating that no single observation was both high-leverage and disproportionately influential on the fitted model. Together, these diagnostics indicate that the regression results are not attributable to a small number of unduly influential cases.

Residual diagnostics revealed no substantial violations of OLS assumptions. Residuals appeared approximately normally distributed, as indicated by inspection of the standardized residual histogram (Figure 4), and residuals-versus-fitted plots showed no systematic patterns suggestive of heteroscedasticity (Figure 5). While the low VIF for FC (1.020) rules out collinearity with other predictors as an explanation for its dominant effect, it does not, on its own, rule out conceptual overlap between the FC and BI item sets, or common-method variance arising from collecting all constructs via a single self-report instrument at a single timepoint. A comparison of item wording (Multimedia Appendix 3) shows the 2 construct sets to be conceptually distinct. FC items ask about resources, knowledge, compatibility, and support, while BI items ask about future usage, recommendation, and reliance, but we cannot rule out common-method bias as a contributor to the magnitude of the observed association given the single-source, single-timepoint design. We formally examined this possibility using Harman’s single-factor test, entering all 28 items across the 7 constructs into an unrotated principal component analysis. Seven components had eigenvalues greater than 1.0, consistent with the intended 7-construct structure, and the first, largest unrotated factor accounted for only 16.0% of total variance, well below the 50% threshold conventionally used to flag a common-method-variance concern. This result indicates that common-method bias is unlikely to be a major contributor to the magnitude of the FC -BI association; the Fornell-Larcker discriminant-validity violation between these 2 constructs reported in the Reliability Analysis section, rather than common-method variance, is therefore the more likely explanation for the strength of this association.

**Figure 4.**
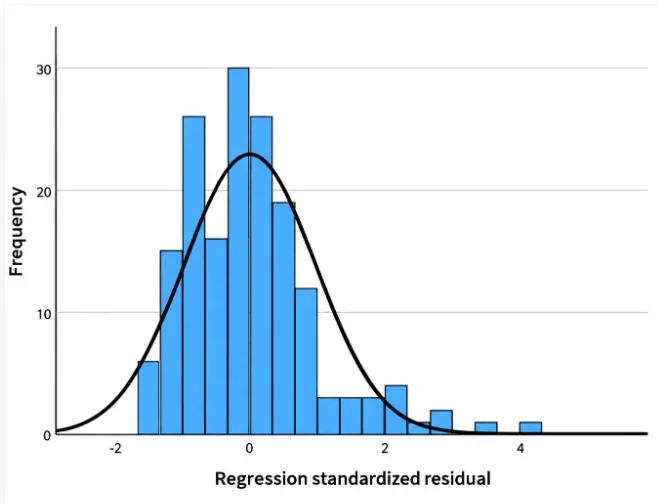
Histogram of standardized residuals for the OLS regression model. OLS: ordinary least squares.

**Figure 5.**
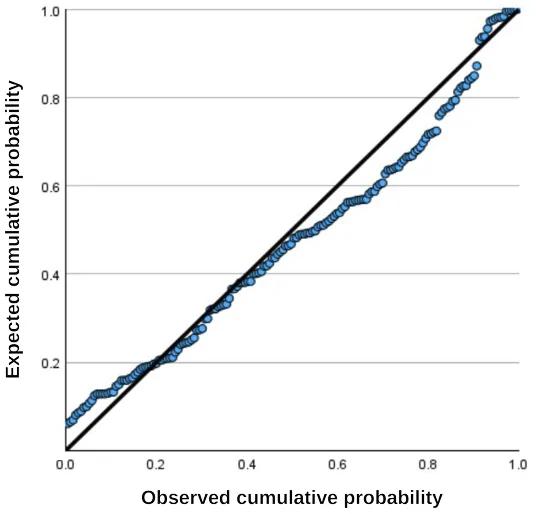
Normal probability-probability plot of regression standardized residual.

### Moderation Analysis

Using the 8 interaction models described in the Methods section, results indicated no significant moderation. All interaction coefficients had *P*-values exceeding 0.10. For example, the Age × FC interaction yielded β=−.002 (SE=.009, *P*=.83) (Table 5), suggesting that FC’s strong positive effect on BI operates equivalently across younger and older participants. Similar nonsignificant patterns emerged for all other interactions. H7 and H8 were therefore not supported: neither age nor education moderated the relationship between any UTAUT construct and BI. This is distinct from the significant main effect of age on BI reported in the Regression Analysis section (β=.105; *P*=.03); that finding indicates older participants reported somewhat higher intention overall, not that age altered the strength of any UTAUT relationship, and the 2 findings should not be conflated.

**Table 5.** Interaction effects of age and education on UTAUT^a^ constructs predicting ^b^BI.

Interaction term	β₃	SE	*t* test (*df*)	*P* values	Interpretation
PE^c^×Age	.008	.012	0.67 (158)	.51	Not significant
EE^d^×Age	−.003	.011	−0.27 (158)	.79	Not significant
SI^e^×Age	.015	.013	1.15 (158)	.25	Not significant
FC^f^ ×Age	−.002	.009	−0.22 (158)	.83	Not significant
PE^g^×Education	−.021	.031	−0.68 (158)	.50	Not significant
EE×Education	.018	.028	0.64 (158)	.52	Not significant
SI×Education	−.012	.030	−0.40 (158)	.69	Not significant
FC×Education	.007	.025	0.28 (158)	.78	Not significant

a UTAUT: Unified Theory of Acceptance and Use of Technology.

b BI: behavioral intention.

c PE: performance expectancy.

d EE: effort expectancy.

e SI: social influence.

f FC: facilitating conditions.

g PE: perceived stigma.

### Qualitative Findings

Table 1 presents the key themes identified from the qualitative feedback, categorized using the approach described in the Categorization of Qualitative Data subsection of the Methods section. Open-ended responses were collected for 3 survey questions (Table 1). Of the total sample (n=168), 168 participants responded to Question 1 (things liked about mHealth apps); 162 participants responded to Question 2 (things disliked about mHealth apps); and 157 participants responded to Question 3 (what would make TechAids more useful for people living with HIV and AIDS). Not all participants provided feedback to every open-ended question.

Several aspects the users appreciate about using mobile apps for consultation and medication delivery were highlighted. The users value the ability to reach health care providers anytime in terms of accessibility and convenience. It will afford them the opportunity to manage care from their preferred location. Features like reminders for appointments and medication intake are highly appreciated through personalization and control on the app. Users feel secure through strong end-to-end privacy and security measures put in place. Timely alerts and easy access to prescriptions help users adhere to their medication intake and stay on track with their treatment. Users are empowered to better understand and manage their health condition through access to reliable health information and education on the app. Communication and support roles leave users feeling supported and informed, especially in managing their ongoing care needs (Figure 6).

**Figure 6.**
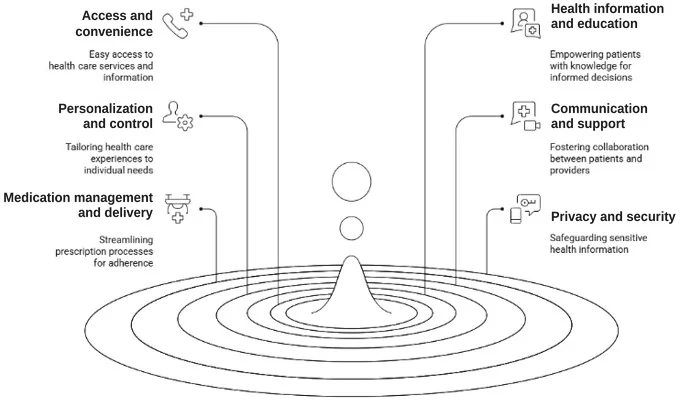
Users' perceived benefits of a mobile app-based platform for consultation and medication delivery

Four key areas of dissatisfaction were identified by users of mobile apps for consultation and medication delivery. User experience during face-to-face consultation is always limited to the allotted time so as to give other opportunities to be attended to. This will only allow limited interaction time and a lack of personal connection with health care providers, leaving users feeling unheard and unsatisfied. Users submitted that poor technical performance and app instability, including frequent crashes, freezing, lagging, and delayed responses disrupt smooth usage and undermine trust in the app. Another major issue pointed out is access to specialists and services, as users often face long waiting times and difficulty finding relevant HIV and AIDS expertise. Dependence on internet connectivity was identified as a significant barrier, as the system will record failure in the use of the app without internet connectivity. The users in low-network or rural areas will find it difficult to receive timely care and support.

The survey findings suggest 8 key areas of improvement to make the mobile app more useful for MSM and transgender individuals living with HIV/AIDS in Nigeria. Users want offline functionality and free internet, which will allow them to use essential features without data or network glitches. Privacy and data security are essential as users expect their sensitive information to be well secured and protected. There is a strong demand for quick helpline support and access to health care providers at any point in time, most especially during emergencies. They also need health education and engagement through regularly updated app content and smart reminders (Figure 7).

**Figure 7.**
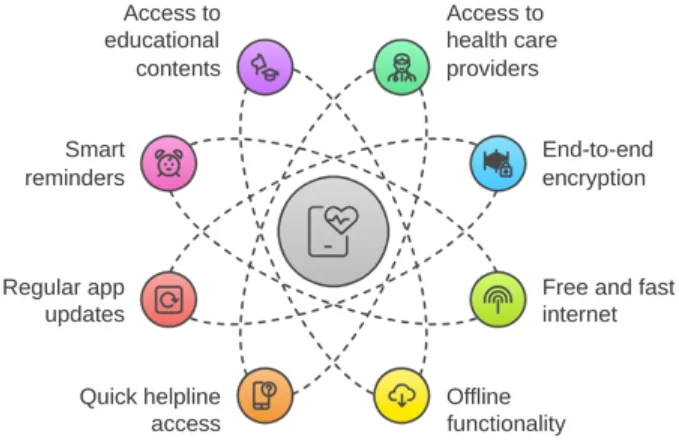
Top recommendations to make TechAids app more useful (authors compilation).

## Discussion

### Principal Findings

This study applied an extended UTAUT model to understand acceptance and intended use of TechAids among MSM and transgender individuals in Nigeria. Using construct-level data and a detailed regression specification, we produced a robust mathematical model of BI and visualized the relative importance of different predictors. The results converge on a clear message that FC is the dominant driver of intention to adopt TechAids. When respondents perceive that they have the necessary resources, infrastructure, and support, they are highly likely to intend to use the platform; when these conditions are absent or fragile, even strong beliefs about usefulness and ease of use do not translate into intention. The nonsignificance of PE, EE, and SI contrasts with much of the prior UTAUT and mHealth literature, warrants more critical interpretation than setting alone can provide. Three contextual explanations, not mutually exclusive, are worth distinguishing. First, infrastructural dominance may be statistically suppressing the explanatory contribution of other predictors: with FC explaining the large majority of variance in intention (*r*=0.802 with BI), the remaining variance available for PE, EE, and SI to predict may simply be too small to detect with this sample size, even if these constructs retain genuine, smaller, theoretical importance. Second, measurement-related explanations are plausible: high nondisclosure rates on sensitive items may have restricted variability not only in trust and PS but, indirectly, in the broader pattern of responses, attenuating observed associations. Third, sample-characteristic explanations are relevant given that the analytic sample combined platform users and health care providers with potentially different bases for evaluating usefulness, ease of use, and social influence. This was addressed directly in the Regression Analysis section via a user-only sensitivity check. We do not interpret these findings as evidence that PE, EE, and SI are unimportant in principle, but rather that, in this resource-constrained and highly stigmatized setting, their influence may be empirically overshadowed by more immediate infrastructural and measurement constraints.

The prominence of FC is consistent with findings from other developing-country contexts where access to devices, connectivity, and support is uneven [28]. In such settings, abstract beliefs about usefulness can be easily overridden by concrete constraints: intermittent data, shared phones, unreliable electricity, or lack of technical help. Our FC items capture perceptions of device availability, internet access, compatibility, and support, all of which proved critical for respondents considering a digital HIV app. From an implementation perspective, this means that digital health tools cannot be divorced from their infrastructural environment. The small but significant effect of age suggests that older respondents within the 21‐40 year range are modestly more inclined to adopt TechAids. Older participants may have longer histories of engagement with HIV care and may view digital tools as a pragmatic way to manage frequent appointments and medication refills. This finding echoes broader work on technology adoption in households, where life cycle and accumulated responsibilities often shape perceived benefits [33].

Trust and PS did not emerge as significant predictors of BI in the regression model, but this should not be taken to mean that they are unimportant. Qualitative work and broader HIV literature emphasize that trust and stigma are central to health-seeking behavior among MSM and transgender communities [34,35]. Qualitatively, users explicitly valued “Privacy & Security” as a feature they liked about consultation and medication-delivery apps, and identified “End-to-end encryption” as a top recommendation for making TechAids more useful (Table 1), both directly relevant to trust and PS. These concerns surfaced spontaneously and prominently in open-ended responses, while the corresponding quantitative constructs were not significant predictors of intention, suggesting this is a measurement and variance issue rather than evidence that trust and stigma are genuinely unimportant to this population. Several factors plausibly explain the gap: high rates of nondisclosure on sensitive identity and HIV-status items (Participant Characteristics) likely restricted the variability available to detect trust and PS effects in the regression model; and because recruitment proceeded through trusted NGO gatekeepers under assured confidentiality conditions (Participants and Recruitment section), participants who agreed to take part may represent a subgroup with comparatively higher baseline trust and lower perceived risk than the broader key population, further compressing variance on these constructs.

For designers, implementers, and policymakers working on digital health for key populations in low-resource settings, the findings point to several priorities:

1. Invest in FC, not just app design: device support, zero, or low-rated data packages, offline functionality, and local helpdesks or digital navigators should be treated as core components of the intervention, not optional extras.
2. Design for infrastructural fragility: features such as low-bandwidth modes, delayed synchronization, SMS fallbacks, and robust error-handling can ensure continuity of care even when connectivity is intermittent.
3. Interpret the age effect cautiously: age showed a small but significant positive main effect on BI, and older participants in our sample expressed modestly higher adoption intention overall. However, moderation analysis found no evidence that age altered the strength of any UTAUT relationship. The data therefore do not support differentiated, age-targeted UTAUT-based messaging strategies, and any age-related design choices should be informed by further targeted research rather than this finding alone.

### Limitation and Future Research

Beyond methodological considerations already discussed, several substantive limitations warrant acknowledgment. Our study examined a single country context (Nigeria) which may not be generalizable to other developing or developed contexts where infrastructural, cultural, or policy conditions differ. Small sample size (N=168) is another limitation of this study which may restrict the statistical power of the analysis and the ability to detect subtle relationships among the UTAUT constructs. Future research could extend this work by collecting larger, multisite samples to strengthen the robustness and combining longitudinal usage data from the deployed TechAids platform with survey measures. The cross-sectional design precludes causal inference, and BI may not reflect sustained real-world use. Analytically, OLS regression of composite Likert constructs should be supplemented by SEM or ordinal regression. AVE fell below the 0.50 threshold for 3 constructs, leaving convergent validity unconfirmed, and a discriminant-validity concern exists between FC and BI. Full Confirmatory Factor Analysis was precluded by sample size. Although Harman’s test found no pervasive common-method bias, single-instrument, single-timepoint measurement cannot fully rule it out; temporally separated measurement of key constructs is recommended.

Combining platform users (n=141) and providers (n=27) in 1 model limits subgroup comparisons; a larger provider sample is needed for stratified analysis. High nondisclosure rates on gender, sexual orientation, and HIV status (33%‐49%) constrain confidence in sample representativeness. Qualitative coding by a single author (EOO) without interrater reliability checks limits robustness. Selection bias through NGO and clinic gatekeepers likely underrepresents the most marginalized individuals, while social desirability bias may affect sensitive disclosures. Finally, use of a prototype rather than the live platform may not capture real-world barriers or sustained engagement challenges.

### Conclusion

This study examined factors predicting intention to adopt TechAids, a digital HIV care platform, among MSM and transgender individuals in Nigeria. Using an extended UTAUT framework, we found that FC, perceptions of internet access, device compatibility, technical knowledge, and support availability emerged as the overwhelmingly dominant predictor of adoption intentions, explaining approximately 65% of variance. Traditional UTAUT constructs (PE, EE, SI) and our theoretical extensions (trust, PS) showed no significant effects, though qualitative data suggested these factors remain conceptually important.

These findings challenge assumptions underlying much digital health discourse. Technology acceptance discussions frequently emphasize cognitive factors, usefulness, ease of use, features, treating infrastructure as merely enabling background conditions. Our results suggest that in resource-constrained settings, this hierarchy inverts: infrastructure becomes foreground, overwhelming cognitive considerations. Well-designed platforms with privacy-enhancing features may generate enthusiasm among potential users, but this enthusiasm translates into adoption only when individuals possess reliable connectivity, adequate devices, and confidence they can access technical support when needed. These findings describe a strong cross-sectional association between perceived FC and BI to adopt TechAids, not a demonstrated causal effect of infrastructure on actual platform use or downstream health outcomes. FC being strongly associated with intention does not, on its own, prove that infrastructure alone determines whether people go on to use the platform or experience improved HIV care outcomes; verifying that pathway requires the longitudinal and real-world usage data called for in Limitation and Future Research section.

For digital health implementers, these findings carry clear implications: infrastructure support must be treated as core intervention components, not optional supplements. Zero-rated data plans, offline functionality, low-bandwidth optimization, device loan programs, and community-based technical support infrastructure should be budgeted alongside platform development. Without these enabling foundations, even the most thoughtfully designed platforms will fail to achieve meaningful adoption and sustained engagement.

## Supplementary material

10.2196/93129 Multimedia Appendix 1 Research hypotheses and assumptions.

10.2196/93129 Multimedia Appendix 2 Demographics table.

10.2196/93129 Multimedia Appendix 3 Reliability and average variance extracted (AVE) by construct.

## Abbreviations

AVE: average variance extracted
BI: behavioral intention
CR: composite reliability
EE: effort expectancy
FC: facilitating conditions
HOHSRI: Happiest Ones Health Support and Rights Initiative
LGBTQ+: lesbian, gay, bisexual, transgender, queer, and other sexual and gender minorities
mHealth: mobile health
MSM: men who have sex with men
NGO: nongovernmental organization
OLS: ordinary least squares
PE: performance expectancy
PS: perceived stigma
SEM: structural equation modeling
SI: social influence
SPSS: Statistical Package for the Social Sciences
UTAUT: Unified Theory of Acceptance and Use of Technology
VIF: variance inflation factor
