# Supplementary material for: Evaluating Acceptance and Use of Digital Health Platforms for Consultation and Medication Delivery Among Men Who Have Sex With Men and Transgender People Living With HIV: Cross-Sectional Survey Informed by an Extended UTAUT Approach
Source: JMIR Hum Factors. 2026 Oct 6;13:e93129. doi: 10.2196/93129 (PMC13641318; doi:10.2196/93129)
Supplement: Supplementary file 1 [file humanfactors-v13-e93129-s001.docx]

**Research Hypotheses and Assumptions**

| **Constructs** | **Hypothesis** | **Assumption** |
| --- | --- | --- |
| Performance Expectancy (PE) | **H1:** Performance expectancy is positively related to the behavioural intention to use and accept TechAids app for consultation and medication delivery among MSM and transgender people living with HIV/AIDS. | **Assumption:** MSM and transgender people living with HIV/AIDS are more likely to use TechAids app if they believe that the platform will help them improve their health and manage HIV effectively. |
| Effort Expectancy (EE) | **H2:** Effort expectancy is positively related to the behavioural intention to use and accept digital health platforms for consultation and medication delivery among MSM and transgender people living with HIV/AIDS. | **Assumption:** When the platform is perceived as easy to use, it reduces user resistance and enhances adoption, especially among users with limited digital literacy. |
| Social Influence (SI) | **H3:** Social inﬂuence has a positive effect on the behavioural intention to use and accept digital health platforms among MSM and transgender people living with HIV/AIDS. | **Assumption:** Peer groups, community advocates, and healthcare providers can encourage use, especially in stigmatized or marginalized communities where trust is critical. |
| Facilitating Conditions (FC) | **H4:** Facilitating conditions positively influence the behavioural intention to use and accept digital health platforms among MSM and transgender people living with HIV/AIDS. | **Assumption:** Access to internet, smartphones, community tech support, and delivery services enhance actual system usage. |
| Perceived Stigma (PS) | **H5:** Perceived Stigma has a negative effect on Behavioural Intention to adopt the TechAids platform. | **Assumption:** Individuals who perceive high levels of stigma or fear of discrimination are less likely to trust or engage with digital-health systems such as TechAids. Reducing stigma through confidentiality assurances, anonymous service options, and supportive communication will therefore increase users’ comfort and intention to adopt TechAids. |
| Trust | **H6:** Trust has a positive effect on Behavioural Intention to adopt the TechAids platform. | **Assumption:** When users perceive TechAids as reliable, secure, and respectful of their privacy, their confidence in the system increases - leading to stronger behavioural intention to use it for consultation, appointment, and medication delivery services. Building transparent data-handling processes and consistent system performance is assumed to enhance this trust - intention relationship. |
| Age | **H7:** Age moderates the relationship between UTAUT constructs and behavioural intention. | **Assumption:** Younger users may respond more strongly to effort expectancy and social influence, while older users may prioritize performance expectancy and facilitating conditions. |
| Education | **H8:** Education moderates the relationships between UTAUT constructs and behavioural intention. | **Assumption:** Higher educational attainment may amplify the influence of cognitive factors (performance expectancy) while reducing the impact of effort expectancy. |
