# Supplementary material for: Evaluating Acceptance and Use of Digital Health Platforms for Consultation and Medication Delivery Among Men Who Have Sex With Men and Transgender People Living With HIV: Cross-Sectional Survey Informed by an Extended UTAUT Approach
Source: JMIR Hum Factors. 2026 Oct 6;13:e93129. doi: 10.2196/93129 (PMC13641318; doi:10.2196/93129)
Supplement: Supplementary file 2 [file humanfactors-v13-e93129-s002.docx]

**Demographics table**

| **Variable** | **Patient/Client N=141** | **Healthcare Professionals  N=27** |
| --- | --- | --- |
| **Education Level** |  |  |
| Master’s Degree | 48(34.0%) | 9(33.3%) |
| Bachelor’s Degree | 43(30.5%) | 9(33.3%) |
| Diploma | 50(35.5%) | 9(33.3%) |
| **Age** |  |  |
| 21-30 | 72(51.1%) | 8(29.6%) |
| 31-40 | 69(48.9%) | 19(70.4%) |
| **Gender** |  |  |
| Male | 44(31.2%) | 10(37.0%) |
| Female | 51(36.2%) | 7(25.9%) |
| Prefer not to say | 46(32.6%) | 10(37.0%) |
| **Gender at Birth** |  |  |
| Yes | 77(54.6%) | 10(37.0%) |
| No | 1(0.7%) | - |
| Does not want to disclose | 63(44.7%) | 17(63.0%) |
| **Identify as Trans** |  |  |
| Yes | 2(1.4%) | - |
| No | 73(51.8%) | 17(63.0%) |
| Prefer not to say | 66(46.8%) | 10(37.0%) |
| **Sexual Orientation** |  |  |
| Gay or Lesbian | 2(1.4%) | - |
| Straight/Heterosexual | 70(49.6%) | 14(51.9%) |
| Prefer not to say | 69(48.9%) | 13(48.1%) |
| **HIV Status** |  |  |
| Negative | 77(54.6) | 14(51.9) |
| Does not want to disclose | 64(45.4) | 13(48.1) |
