# Supplementary material for: Evaluating Acceptance and Use of Digital Health Platforms for Consultation and Medication Delivery Among Men Who Have Sex With Men and Transgender People Living With HIV: Cross-Sectional Survey Informed by an Extended UTAUT Approach
Source: JMIR Hum Factors. 2026 Oct 6;13:e93129. doi: 10.2196/93129 (PMC13641318; doi:10.2196/93129)
Supplement: Supplementary file 3 [file humanfactors-v13-e93129-s003.docx]

**Reliability and Average Variance Extracted (AVE) by construct *(AVE values below the 0.50 convergent-validity threshold are italicised)***

| **Constructs** | **Factor Loads** | **No. of Items** | **Cronbach’s alpha (α)** | **CR** | **AVE** | **√AVE** |
| --- | --- | --- | --- | --- | --- | --- |
| **Performance Expectancy** |  |  |  |  |  |  |
| **PE1**. Using TechAids App would improve my wellbeing | 0.762 | 4 | 0.733 | 0.748 | *0.429* | 0.665 |
| **PE2**. I find TechAids App useful in my daily activities | 0.736 |  |  |  |  |  |
| **PE3**. Using TechAids App would increase my productivity | 0.715 |  |  |  |  |  |
| **PE4**. Using TechAids App would help me achieve my tasks more efficiently | 0.801 |  |  |  |  |  |
| **Effort Expectancy** |  |  |  |  |  |  |
| **EE1**. Learning how to use TechAids App is easy for me | 0.752 | 4 | 0.768 | 0.770 | *0.457* | 0.676 |
| **EE2**. I find TechAids App simple to use | 0.715 |  |  |  |  |  |
| **EE3**. I can use TechAids App without significant effort | 0.816 |  |  |  |  |  |
| **EE4**. I found it easy to get TechAids App to do what I want it to do | 0.786 |  |  |  |  |  |
| **Social Influence** |  |  |  |  |  |  |
| **SI1**. People who influence my behaviour think I should use TechAids App | 0.831 | 4 | 0.796 | 0.802 | 0.509 | 0.713 |
| **SI2**. People whose opinions I value prefer that I use TechAids App | 0.798 |  |  |  |  |  |
| **SI3**. My peers or colleagues support the use of TechAids App | 0.668 |  |  |  |  |  |
| **SI4**. I feel pressure to use TechAids App from those around me | 0.849 |  |  |  |  |  |
| **Facilitating Conditions** |  |  |  |  |  |  |
| **FC1**. I have the resources necessary to use TechAids App | 0.753 | 4 | 0.831 | 0.835 | 0.561 | 0.749 |
| **FC2**. I have the knowledge necessary to use TechAids App | 0.848 |  |  |  |  |  |
| **FC3**. TechAids App is compatible with the tools and systems I use | 0.835 |  |  |  |  |  |
| **FC4**. There is adequate support available to help me use TechAids App | 0.826 |  |  |  |  |  |
| **Trust** |  |  |  |  |  |  |
| **TR1** I believe TechAids securely protects my personal and health information | 0.791 | 4 | 0.887 | 0.889 | 0.669 | 0.818 |
| **TR2** I trust TechAids to keep my HIV-related information confidential | 0.893 |  |  |  |  |  |
| **TR3** I believe TechAids is reliable for managing my health needs | 0.903 |  |  |  |  |  |
| **TR4** I feel confident that TechAids acts in my best interest when delivering healthcare services | 0.867 |  |  |  |  |  |
| **Perceived Stigma** |  | 4 | 0.842 | 0.842 | 0.572 | 0.756 |
| **PS1** I am worried that using TechAids could expose my HIV status to others | 0.820 |  |  |  |  |  |
| **PS2** I am concerned that people might judge me if they know I use TechAids. | 0.821 |  |  |  |  |  |
| **PS3** I fear discrimination if my use of TechAids becomes known | 0.831 |  |  |  |  |  |
| **PS4** Concerns about stigma make me hesitant to use TechAids | 0.823 |  |  |  |  |  |
| **Behavioural Intention** |  | 4 | 0.701 | 0.707 | *0.382* | 0.618 |
| **BI1**. I intend to use TechAids App regularly in the future | 0.789 |  |  |  |  |  |
| **BI2**. I plan to continue using TechAids App whenever needed | 0.688 |  |  |  |  |  |
| **BI3**. I will recommend the use of TechAids App to others | 0.702 |  |  |  |  |  |
| **BI4**. I am likely to depend on using TechAids App for my tasks | 0.729 |  |  |  |  |  |
